# Epistasis decreases with increasing antibiotic pressure but not temperature

**DOI:** 10.1098/rstb.2022.0058

**Published:** 2023-05-22

**Authors:** Ana-Hermina Ghenu, André Amado, Isabel Gordo, Claudia Bank

**Affiliations:** ^1^ Instituto Gulbenkian de Ciência, Rua da Quinta Grande 6, Oeiras 2780-156, Portugal; ^2^ Division of Theoretical Ecology and Evolution, Institut für Ökologie und Evolution, Universität Bern, Baltzerstrasse 6, 3012 Bern, Switzerland; ^3^ Swiss Institute of Bioinformatics, 1015 Lausanne, Switzerland

**Keywords:** environmental interactions, cryptic epistasis, RNA polymerase B (*rpoB*), genetic interactions, rifampicin

## Abstract

Predicting mutational effects is essential for the control of antibiotic resistance (ABR). Predictions are difficult when there are strong genotype-by-environment (G × E), gene-by-gene (G × G or epistatic) or gene-by-gene-by-environment (G × G × E) interactions. We quantified G × G × E effects in *Escherichia coli* across environmental gradients. We created intergenic fitness landscapes using gene knock-outs and single-nucleotide ABR mutations previously identified to vary in the extent of G × E effects in our environments of interest. Then, we measured competitive fitness across a complete combinatorial set of temperature and antibiotic dosage gradients. In this way, we assessed the predictability of 15 fitness landscapes across 12 different but related environments. We found G × G interactions and rugged fitness landscapes in the absence of antibiotic, but as antibiotic concentration increased, the fitness effects of ABR genotypes quickly overshadowed those of gene knock-outs, and the landscapes became smoother. Our work reiterates that some single mutants, like those conferring resistance or susceptibility to antibiotics, have consistent effects across genetic backgrounds in stressful environments. Thus, although epistasis may reduce the predictability of evolution in benign environments, evolution may be more predictable in adverse environments.

This article is part of the theme issue ‘Interdisciplinary approaches to predicting evolutionary biology’.

## 1. Introduction

It has been debated for decades whether gene-by-gene interactions (G × G or epistasis) influence evolutionary processes (reviewed in [1,2]). Compelling evidence for the successive fixation of epistatic substitutions comes from studies of amino acid replacements over phylogenetic time scales (e.g. [3–5]). Moreover, experimental evolution has demonstrated that adaptive paths can be constrained by epistasis (e.g. [6]). Also, the environment can modulate mutational effects through gene-by-environment (G × E) interactions. Whereas multiple studies have demonstrated the existence of G × E interactions (e.g. [7–9]), our knowledge of the extent and the consequences of G × G × E interactions, i.e. those in which epistasis interacts with the environment, are limited ([10–13], reviewed in [2]).

G × G, G × E and G × G × E interactions complicate evolutionary predictions because they alter expected phenotypes or fitness of individuals [2,14]. Studying their extent and incorporating their effect into evolutionary predictions is daunted by the complexity of the genotype space and the myriad of environments that could be tested [15]. However, our lack of quantitative knowledge of G × G and G × E effects poses dangers to fields in which genetic and evolutionary models are applied: they could lead to the failed genetic rescue of endangered species, or the unexpected spread or maintenance of antibiotic resistance (ABR).

At the same time, the existence of G × E interactions is a crucial assumption in ABR evolution. By definition, the original genotype grows well in the antibiotic-free environment and poorly in the antibiotic environment. Conversely, a resistant genotype grows well in the antibiotic environment and is usually assumed to have low fitness in the antibiotic-free environment, resulting in so-called costs of ABR (reviewed in [16], but see [17]). In addition, G × E interactions with other environmental variables, such as high temperature or minimal media, can facilitate the evolution of resistant genotypes in the absence of antibiotics [6,18]. Although G × E interactions are central in ABR evolution, many questions about the role of G × E remain unexplored (but see [19–21]); for example, how does the G × E relationship between susceptible and resistant genotypes change across non-antimicrobial environmental axes, and how frequently is ABR modulated by the genetic background (i.e. through G × G effects).

ABR was first identified as a problem shortly after the introduction of antibiotics for clinical use [22], and it continues to be a leading cause of death worldwide [23]. For this reason, inferring potential resistances, cross-resistances, compensatory mutations and, ultimately, predicting the evolutionary trajectories of bacterial genomes in the presence of antibiotics is an application of evolutionary biology that is important for the health of humans and the planet [24,25]. Critically, G × G and G × E effects influencing ABR can complicate the identification of new resistances. For example, with G × G effects, resistance may depend on more than one easily identifiable allele. In addition, with G × E effects, laboratory evolution settings may not be informative of the fitness of genotypes in the wild.

Including G × G effects in the study of ABR requires the consideration of many potentially interacting genotypes. With epistasis, interactions can be specific between particular mutations due to mechanistic interactions of residues or proteins, for example (reviewed in [26]), or global, where any combination of mutations ultimately shows a pattern of G × G [27,28]. In contrast to increasing numbers of reports of ubiquitous epistasis [12,29,30], much existing work to date has considered single ABR genotypes at a time under the assumptions of additive effects (but see [31,32]). The theoretical concept of a fitness landscape, which maps every possible genotype to its fitness, captures G × G interactions and enables the study of how such interactions affect evolutionary trajectories (reviewed in [2,33]). Unless there is a known underlying pattern of interactions, epistasis makes evolution on the fitness landscape less predictable. Moreover, with added G × E interactions, the fitness landscape may change across environmental gradients, leading to changes in both single genotypes and epistasis [8,12] and to different possible evolutionary trajectories [13,34].

We here take a step towards quantifying G × G × E interactions by studying 15 small (i.e. two mutational-step) fitness landscapes across two environmental gradients and their combination, antibiotic concentration and temperature. In our fitness landscapes, we combine three known ABR mutations in the *rpoB* gene with five gene knock-outs, resulting in 24 genotypes that are screened for competitive fitness in 12 abiotic environments. Unlike previous studies, we study interactions of two different mutation types, single amino acid substitutions (more specifically, single-nucleotide polymorphisms, or SNPs) and whole gene deletions. By screening the fitness landscapes across a grid of environments, we quantify their change across environmental gradients, which is related to the robustness of fitness predictions under environmental change. Since the ABR mutations and gene knock-outs were selected based on their *not* exhibiting any direct, mechanistic interactions, one might *a priori* expect few G × G interactions and that the fitness of the double mutants would be additive as compared to the single mutants. On the other hand, non-specific epistasis (epistasis between mutations that do not directly interact mechanistically) has been observed ubiquitously [26]. Our fitness landscapes feature moderate epistasis in the benign environments, which decreases at higher antibiotic concentrations. Altogether, we conclude that our fitness landscapes are very predictable across environments.

## 2. Results

### (a) Choice of genotypes

We selected rifampicin-resistant single-nucleotide mutations that were *a priori* known to differ in their performance between different temperature environments in M9 minimal medium. The ABR mutations modify single amino acids at the antibiotic target site in the gene RNA polymerase B (*rpoB*). Prior to our study, it was unknown how the mutants would perform in different combinations of antibiotic and high-temperature environments (i.e. we only knew about the effect of one environmental axis on its own). *RpoB* H526Y was established to show a trade-off between environment types: it confers high rifampicin resistance at 37°C but grows poorly as compared to the wild type at higher temperatures in the absence of antibiotic [7,35]. Conversely, *rpoB* S512F was established to have a synergy between environment types: it grows well both at high rifampicin concentrations and higher temperatures [7], separately. Finally, *rpoB* I572N is a weak rifampicin resistance mutation that was first identified during evolution to high temperature [6,36].

We initially tried to select knock-out mutations similarly as we had done for *rpoB* mutants: we used data from a large phenotypic screen of *Escherichia coli* (*E. coli*; accessed from https://ecoliwiki.org/tools/chemgen/) to select knock-out mutations that were known to differ in their performance between environment types. However, neither the bacterial colony size s-scores [37] nor the bacterial colony opacity and density s-scores [38] were able to predict the growth effects of Keio collection [39] knock-out mutants in batch culture environments (data not shown).

**Figure 1.**
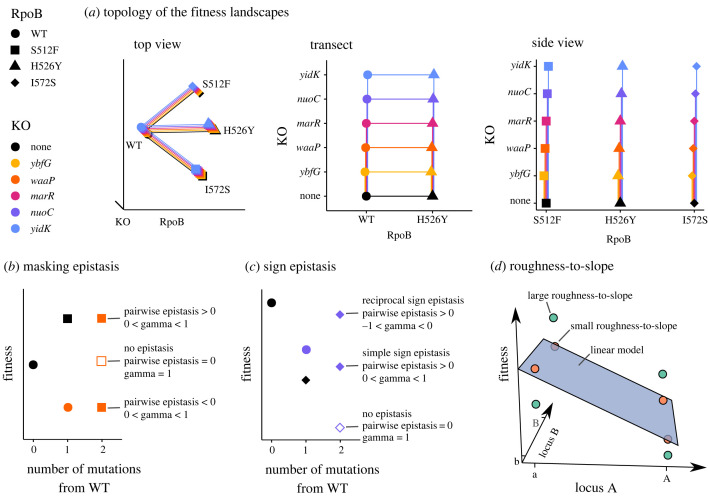
(*a*) Three-dimensional schematics depicting the topology of the studied fitness landscapes. The schematics show the same topology from different perspectives. All mutations are at unique, di-allelic loci. There are 15 fitness landscapes each composed of the wild type (WT), a *rpoB* single mutant, a knock-out (KO) single mutant, and the resultant double mutant. Lines connect genotypes that are one mutational-step apart. Depiction of ‘classical’ pairwise epistasis and gamma epistasis metrics for (*b*) masking and (*c*) sign epistasis. Masking epistasis is when the fitness effect of a beneficial or deleterious mutation on the wild-type background is zero on a different background. Sign epistasis occurs when the beneficial or deleterious effect of a mutation reverses in the presence of another mutation to become deleterious or beneficial, respectively. Reciprocal sign epistasis is a special case of sign epistasis where both mutations change sign on the other’s background. The gamma statistic measures the correlation of fitness effects among the different genetic backgrounds where a mutation appears. The white-filled shape shows the expected fitness value *without* epistasis. For (*a*–*c*), the shape indicates the *rpoB* point mutation and the colour indicates which gene has been knocked-out; the black circle indicates the wild-type genotype without any mutations on *rpoB* and without any knock-outs. (*d*) Fitness landscapes exhibiting large (teal points) and small (orange points) roughness-to-slope ratios. The roughness-to-slope statistic measures how different the fitness landscape is from an additive, linear model (a purely additive landscape has a ratio of zero). The plane shows the fit of the multidimensional linear model to the fitness landscapes for two di-allelic loci. The vertical distance between the genotype (i.e. point) and the plane shows its residual. Epistasis is quantified as the ratio of the standard deviation of the residuals to the mean of the slopes of the plane. Note that (*d*) does not follow the colour and shape scheme of the other panels.

Ultimately, we selected knock-out mutations based on their *a priori* functional effects and the presence of segregating knock-out polymorphism in natural populations (accessed from the panX database at https://pangenome.org/; [40]). We selected four knock-outs of functional genes, all involved in how the cell interacts with its environment as follows: *marR* (multiple ABR regulator) detects and responds to chemical stressors, and its knock-out is sensitive to heat shock [41], conferring either resistance [42] or susceptibility [43] to some antibiotics but not rifampicin [37]; *nuoC* and *yidK* are both located on the plasma membrane and involved in transport [44,45]; and *waaP* impacts outer membrane stability through lipopolysaccharide biosynthesis [46]. The fifth gene, *ybfG*, is a pseudogene and was selected as a control since its knock-out should perform similarly to the wild-type genotype. None of the knock-outs interact directly with *rpoB* or other genes involved in transcription, protein synthesis or DNA supercoiling. Therefore, any epistasis that will be observed should be an instance of ‘non-specific epistasis’ [26]. Non-specific epistasis is attributable to factors *other than* mechanistic interactions: for example, nonlinearities in the genotype-to-phenotype map. Figure 1*a* shows a schematic of the resulting topology of the fitness landscape when combining the ABR and knock-out mutations.

Illumina whole-genome re-sequencing was used to confirm the constructed mutants and identify any *de novo* mutations compared to the wild-type genotype (electronic supplementary material, table S3). See results in electronic supplementary material for details.

### (b) Choice of environments

Since the wild type and knock-out single mutants are sensitive to rifampicin, the screened antibiotic concentrations were selected based on the wild-type dose response (electronic supplementary material, figure S14). The screened temperature environments were selected based on their physiological relevance for *E. coli* and its human host: 37°C is the temperature of a healthy human host, 40°C is the temperature of high fever and 42°C is near the maximum temperature that *E. coli* can withstand. Antibiotic-susceptible genotypes (i.e. genotypes that carry the wild-type allele at the *rpoB* locus) can be considered as ‘specialists’ of antibiotic-free environments because their growth deteriorates in the presence of the antibiotic, i.e. they show strong G × E interactions. Based on previous results, we expected that the *rpoB* S512F single mutant that grows well in the presence of antibiotic and at high temperature, separately, would exhibit ‘generalist’ growth in all environments with little G × E interaction.

### (c) Measure of competitive fitness

The wild type, labelled by Green Fluorescent Protein (GFP), single and double mutants were competed against the same reference genotype, mCherry-labelled *rpoB* H526Y, in all environments by starting at approximately equal ratios and a fixed inoculum size. The competitive index ($\hat{w}$), a proxy for fitness, was estimated after 20 h of batch culture growth as follows:

$$\hat{w}=\ln\left(\frac{D_{\mathrm{comp}}^{f}/D_{\mathrm{ref}}^{f}}{D_{\mathrm{comp}}^{i}/D_{\mathrm{ref}}^{i}}\right),$$

where *D*^*i*^ represents the initial density and *D*^*f*^ represents the final density as measured by flow cytometry. We found nearly identical $\hat{w}$ estimates when the fluorophores of the reference and competitor genotypes were swapped (${\hat{w}}_{\mathrm{GFP}}=\beta \;\cdot \;{\hat{w}}_{\mathrm{mCherry}}+b$; adjusted *R*^2^ = 0.95; *β* = 0.96, *p* < 10^−15^; electronic supplementary material, figure S15 and table S4); including the effect of environment in the regression was not significant (*F*_11,127_ = 1.27, *p* = 0.25; electronic supplementary material, table S5 and figure S16), but adding the effect of genotype was significant (*F*_11,127_ = 4.00, *p* < 10^−4^; electronic supplementary material, figure S17 and table S6), although genotype explains only about 1% more of the variation in the data (adjusted *R*^2^ = 0.96 for overall regression) as compared to not including this effect. Therefore, we concluded that fluorescence is a neutral marker.

**Figure 2.**
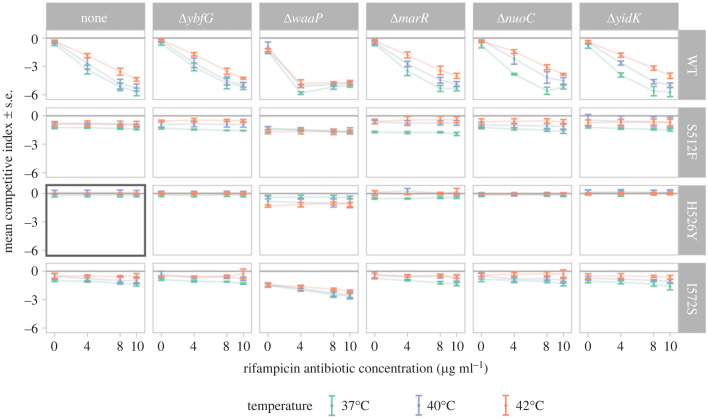
Estimated competitive index ($\hat{w}$) across all genotypes and environments. The *x*-axis shows the four rifampicin antibiotic concentration environments (μg ml^−1^) and the *y*-axis shows the mean estimated competitive index with error-bars for standard errors (*n* = 3–4). Colours show the three temperature environments. Knock-out genotypes are shown in each column (with ‘none’ indicating the wild-type-like state of having no knock-outs) and *rpoB* antibiotic resistant mutants are shown in each row (with ‘WT’ indicating the susceptible wild-type *rpoB* sequence); therefore, the plot on the top left shows the wild type without any mutations and, similarly, the left column shows all *rpoB* single mutants while the top row shows all knock-out single mutants. Finally, the grey box indicates the *rpoB* H526Y genotype used as the reference for all competitions. All experiments were performed in M9 liquid minimal media with 0.4% glucose. The minimum inhibitory concentrations (MIC) for rifampicin (μg ml^−1^) in LB agar medium are as follows: WT ≈ 6 [47], 20 < I572S < 50, S512F > 100 [48], H526Y > 768 [47].

The mean $\hat{w}$ for all genotypes and environments is shown in figure 2. As expected, genotypes that did not have an ABR mutation (figure 2*a*) performed worse than the ABR reference competitor ($\hat{w}<0$) when antibiotic was present. The Δ*waaP* single mutant (figure 2 top right facet) was the most susceptible to the antibiotic of all the investigated genotypes.

For all knock-out backgrounds, we compared the competitive fitness of the ABR mutations against the *rpoB* wild-type sequence in the absence of antibiotic to ascertain whether there was a cost of resistance. We observed no consistent cost of resistance across all backgrounds (electronic supplementary material, figure S18) and (in contrast to previous studies [7,36]) all three ABR mutations grew similarly to the wild type at higher temperatures.

### (d) Multiple summary statistics found few gene-by-gene interactions

Several measures exist to determine the extent of G × G interactions (also termed epistasis). ‘Classical’ pairwise epistasis compares the observed mutational effect of the double mutant against the additive expectation from each of the two single mutants (figure 1*b*,*c*). We inferred pairwise epistasis by using the fitness of the wild-type genotype as a baseline and subtracting the fitness effects of both ABR and knock-out single mutants from the fitness effect of the double mutant (electronic supplementary material, figures S19 and S20 show examples without and with pairwise epistasis, respectively). We estimated pairwise epistasis for each of the 15 sets of double mutations in each of the 12 environments (electronic supplementary material, figure S21). We found significant pairwise epistasis only for the Δ*waaP* mutant. Across all ABR backgrounds, the Δ*waaP* mutant achieved a maximum value of positive pairwise epistasis at the low antibiotic concentration (4 μg ml^−1^). In the absence of antibiotic and at higher antibiotic concentrations (8 − 10 μg ml^−1^), the Δ*waaP* mutant exhibited no significant pairwise epistasis on most of the ABR backgrounds (small negative pairwise epistasis was observed with some ABR backgrounds). This dose-dependent peak of the pairwise epistasis arises because the Δ*waaP* single mutant has a large deleterious fitness effect at 4 μg ml^−1^ and, when this knock-out is found in combination with an ABR mutation, the ABR mutation rescues bacterial growth (e.g. electronic supplementary material, figure S20 second column). We detected almost no pairwise interactions of the Δ*waaP* mutant at higher antibiotic concentrations because the relative fitness effect of the Δ*waaP* mutation becomes much smaller as compared to the fitness effect of the ABR mutations (e.g. electronic supplementary material, figure S20 third and fourth columns).

**Figure 3.**
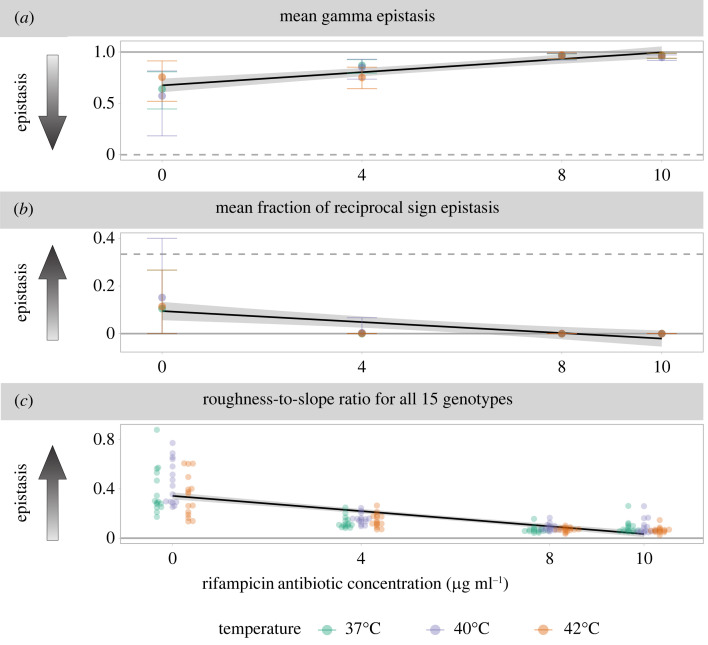
Antibiotic concentration has a significant negative trend with three measures of epistasis: (*a*) the mean gamma epistasis across all loci, (*b*) the mean fraction of loci exhibiting reciprocal sign epistasis and (*c*) the roughness-to-slope ratios by genotype. For each panel the *x*-axis shows the rifampicin antibiotic concentration in μg ml^−1^, the colours show the temperature, and the *y*-axis shows the value of the epistasis measure. Arrows indicate the direction of stronger epistasis, solid horizontal lines show values of no epistasis, and dashed horizontal lines show the values of the maximum possible epistasis for each metric. The black trend lines show the significant negative correlations between antibiotic concentration and each epistasis measure, and the grey shaded regions show the 95% confidence interval of the corresponding least-squares estimated linear regression. For (*a*,*b*), the error bars show the 95% parametric bootstrap confidence intervals. For figure clarity, bootstrap confidence intervals are omitted in (*c*) but can be found in electronic supplementary material, figure S24, which shows the roughness-to-slope ratios for individual genotypes and environments. Significance is reported at the level *α* = 0.01.

Second, we inferred epistasis over the whole landscape using the gamma statistic [49], which measures the correlation of fitness effects among the different genetic backgrounds where a mutation appears (figure 1*b*,*c*). Mutations that exhibit little G × G interaction have similar effects on different backgrounds; consequently, their effects will be strongly correlated across backgrounds (gamma near one). On the other hand, a weak or negative correlation of the effects of a mutation in different backgrounds (gamma near zero or negative, respectively) indicates that a mutation exhibits G × G interactions. Overall, we observed gamma epistasis values near one, which are indicative of very little G × G interaction. Antibiotic concentration was positively correlated with gamma epistasis (figure 3*a*; *F*_1,10_ = 52.7, *p* < 10^−4^, adjusted *R*^2^ = 0.825) regardless of temperature. This means that the strength of G × G interactions decreased as antibiotic concentration increased and that it was independent of temperature. Focusing solely on the gamma epistasis of ABR mutants or knock-outs, respectively (electronic supplementary material, figure S22), we observed different qualitative but non-significant trends between the mutational classes and between the different temperatures. ABR mutations exhibited the strongest epistasis (i.e. gamma near zero) at the intermediate antibiotic concentration and increased with temperature. Conversely, knock-out mutations exhibited the strongest epistasis (again, gamma near zero) at the two highest antibiotic concentrations and decreased with temperature. Independent of the focal mutational class, there was less gamma epistasis at 42°C than at 37°C.

Third, we inferred the presence of simple sign and reciprocal sign epistasis (figure 1*c*). We identified simple sign epistasis as instances where the sign of the fitness effect of a mutation changed depending on its background in over five per cent of bootstrap samples. Then we identified reciprocal sign epistasis as instances where two mutations show sign epistasis on each other’s background. Importantly, the presence of reciprocal sign epistasis is a prerequisite for multiple peaks in the fitness landscape [50]. In our study, significant reciprocal sign epistasis occurred only in antibiotic-free environments but across all temperatures. This trend can be summarized as a negative correlation between antibiotic concentration and the mean fraction of reciprocal sign epistasis (figure 3*b*; *F*_1,10_ = 20.3, *p* < 0.005, adjusted *R*^2^ = 0.637). Reciprocal sign epistasis was observed between the weak ABR mutation *rpoB* I572S and most of the knock-out mutations (electronic supplementary material, table S7). Apart from that, the fraction of simple sign epistasis was significant in all environments and showed no trend across environments (*F*_1,10_ = 2.15, *p* = 0.17; electronic supplementary material, figure S23).

Next, we computed the roughness-to-slope ratio. The roughness-to-slope ratio fits a linear model to the fitness landscape and then quantifies epistasis as the ratio of the standard deviation of the residuals to the linear component of the fit (figure 1*d*). Thus, completely additive landscapes exhibit a roughness-to-slope ratio of zero [50]. In our data, the mean estimated roughness-to-slope ratio was negatively correlated with antibiotic concentration (figure 3*c*; *F*_1,178_ = 206.9, *p* < 10^−15^, adjusted *R*^2^ = 0.535). Roughness-to-slope ratios were very small for all genotypes in the presence of the antibiotic; nevertheless the 95% bootstrap confidence intervals were significantly different from zero for all values.

**Figure 4.**
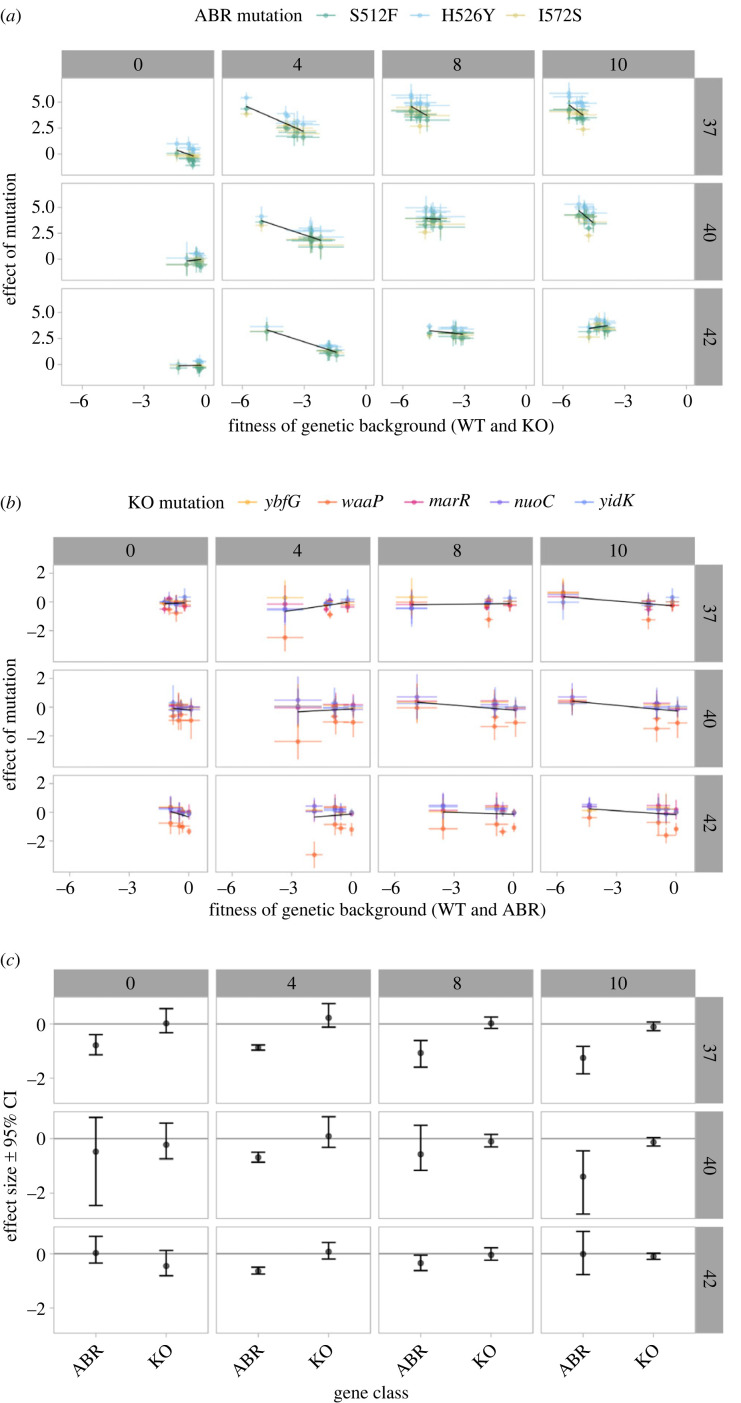
ABR *rpoB* mutations exhibit diminishing-returns epistasis in most environments whereas knock-out mutations never exhibit diminishing-returns epistasis. In each panel, the columns show different rifampicin antibiotic concentrations (in units of μg ml^−1^) and the rows show different temperature environments (in °C). (*a*) ABR *rpoB* mutations exhibit diminishing-returns epistasis in eight of the 12 environments. The *x*-axis shows the fitness $\hat{w}$ of the background (either the wild type or one of the five knock-out mutations) and the *y*-axis shows the effect size of the ABR mutation on that background. (*b*) Knock-out mutations do not exhibit diminishing-returns epistasis in any of the environments. The *x*-axis shows the fitness $\hat{w}$ of the background (either the wild type or one of the three ABR *rpoB* mutations) and the *y*-axis shows the effect size of the knock-out mutation on that background. For (*a*,*b*), the points show mean values and are coloured corresponding to the mutation; the error bars show 95% parametric bootstrap confidence intervals for each value. The black lines show the means of the regressions across all bootstrap samples. (*c*) Quantification of the mean slope effect sizes (±95% confidence intervals) of the diminishing-returns epistasis for the *rpoB* mutations (ABR) and the knock-out mutations (KO). The mean slopes are shown as black trend lines in (*a*) and (*b*). Confidence intervals are determined from linear regressions of each of the parametric bootstrap samples. The confidence intervals that overlap with zero indicate slopes that are *not* significantly different from zero.

Finally, we inferred whether the studied fitness landscapes exhibited diminishing-returns epistasis. Diminishing-returns epistasis measures whether the effect size of a beneficial mutation decreases as the fitness of the genetic background increases and is one of the most frequently observed patterns of epistasis in fitness landscapes [2]. We observed that the fitness effects of the knock-out mutations were not correlated with the overall fitness of the background, regardless of the environment (figure 4*a*,*c*). By contrast, for most environments, the ABR mutations exhibited a negative correlation with the fitness of the background that the mutation is on, indicating a pattern of diminishing-returns epistasis (figure 4*b*,*c*). The observed trend of diminishing returns for the knock-out and ABR mutants did not depend on the low fitness of the Δ*waaP* mutants. Indeed, when the Δ*waaP* backgrounds were removed from the analysis, the trend became more pronounced as significant diminishing-returns epistasis was observed in all but one of the 12 environments (electronic supplementary material, figure S25).

### (e) Competitive fitness is predicted by antibiotic concentration

We used multiple linear regression to study the quantitative effects of antibiotic concentration and temperature environments on the competitive index, $\hat{w}$. We found that the ‘null model’ regression with $\hat{w}$ as the dependent variable and only additive effects of genotype and environment variables was able to explain a considerable amount of the variation observed in the data (adjusted *R*^2^ = 0.68, *F*_13,982_ = 162.5, *p* < 10^−15^; electronic supplementary material, table S8). Most of the residual variation was attributable to the wild-type *rpoB* genotypes (electronic supplementary material, figure S26). Contrary to resistant genotypes, the antibiotic-susceptible genotypes interacted with the rifampicin environments by having higher fitness in the absence of rifampicin but very low fitness at higher rifampicin concentrations.

Next, we quantified the interaction between the *rpoB* antibiotic susceptible and resistant genotypes with the antibiotic concentration by fitting a model with the additive effects and the *G*_*rpoB*_ × *E*_AB_ interaction. This model fitted the data better than the additive null model, explaining almost all of the variation (adjusted *R*^2^ = 0.881, *F*_9,973_ = 186.0, *p* < 10^−15^; electronic supplementary material, figure S27). Polynomial contrasts indicated that higher temperature was correlated with increased $\hat{w}$ (*t* = 15.0, *p* < 10^−15^; electronic supplementary material, figures S28 and S29). The fixed effect of antibiotic concentration alone on $\hat{w}$ was not significant (electronic supplementary material, figure S28). Instead, the effect of antibiotic concentration was only significant as an interaction term with the susceptible *rpoB* wild-type genotype or the weak ABR genotype I572S.

Then, we compared the above model with a slightly more complex model that included an additional *G*_*rpoB*_ × *G*_KO_ interaction between genotypes (i.e. ‘classical’ pairwise epistasis between *rpoB* and knock-out mutations),

$$\hat{w}∼\underset{\mathrm{additive}\;\mathrm{effects}}{\underbrace{G_{rpoB}+G_{\mathrm{KO}}+E_{\mathrm{AB}}+E_{T}}}+\underset{1\mathrm{st}\;\mathrm{order}\;\mathrm{interactions}}{\underbrace{G_{rpoB}\times E_{\mathrm{AB}}+G_{rpoB}\times G_{\mathrm{KO}}}},$$

Although this model with G × E and G × G effects was significantly different from the model with only G × E effects (*F*_15,958_ = 2.60, *p* < 0.001; electronic supplementary material, figure S30), none of the additional coefficients were significant (electronic supplementary material, figure S31), and the model predictions were very similar to those of the model with only G × E. Indeed, the amount of variation explained by the more complex model (adjusted *R*^2^ = 0.883) showed only a 0.2%-point improvement in the total variation explained as compared to the simpler model with only G × E effects. This finding confirms our previous results of small but statistically significant pairwise epistasis in our data.

Despite our relatively large dataset (*n* = 996), we did not have sufficient data to systematically study second-order interactions without overfitting. We fitted models with second-order interactions for descriptive purposes (see electronic supplementary material, results 8.6.3). In brief, we found that there may be a higher-order interaction between epistasis and temperature that our data has insufficient power to reveal statistically.

### (f) Environmental quality is less important for predicting gene-by-gene interactions than antibiotic concentration alone

To study the interactions between epistasis and environment (G × G × E) without the problem of overfitting, we summarized the temperature and antibiotic environments as a single, continuous environmental quality metric that we used to regress the different epistasis measures [51]. We compared three metrics of environmental quality (mean growth of the reference strain, mean growth of all competitor strains, and mean combined growth of reference and competitor strains) and found that the mean growth of all competitor strains in each environment was the best metric of environmental quality (electronic supplementary material, results 8.7.1). This metric of environmental quality was able to distinguish environments from each other (electronic supplementary material, figure S32) and correctly distinguish specialist from generalist genotypes (electronic supplementary material, figure S33). As expected, the environmental quality metric was correlated with antibiotic concentration (adjusted *R*^2^ = 0.559) and somewhat correlated with temperature (adjusted *R*^2^ = 0.325).

We computed the correlation of environmental quality with all of the above measures of G × G by fitting the environmental quality metric as a linear predictor of pairwise epistasis (electronic supplementary material, figures S37 and S38), gamma epistasis (electronic supplementary material, figure S39), the fraction of sign epistasis (electronic supplementary material, figure S40), and the roughness-to-slope ratio (electronic supplementary material, figure S41). Most of the regressions were either not significant to *α* = 0.01 (gamma epistasis for ABR mutations, simple sign epistasis, reciprocal sign epistasis and pairwise epistasis excluding Δ*waaP*) or explained very little of the variation in the data (pairwise epistasis including Δ*waaP*: adjusted *R*^2^ = 0.036). For metrics that exhibited a significant effect of environmental quality on G × G (total gamma epistasis, electronic supplementary material, figure S39; and roughness-to-slope ratio, figure S41), antibiotic concentration alone regressed as a dependent variable explained more of the variation in the G × G metric than was explained by environmental quality. Moreover, the results of the regression on environmental quality were broadly in agreement with our above findings that G × G decreases as antibiotic concentration increases and environmental quality deteriorates (i.e. positive correlations were found between the environmental quality metric and the G × G summary statistics). The only exception was the gamma epistasis for knock-out mutations (electronic supplementary material, figure S39, middle facet), which exhibited the opposite trend to other G × G metrics (i.e. G × G was found to increase as environmental quality deteriorated).

## 3. Discussion

Whether mutational effects and epistasis vary across environments, and how this matters for evolution, has been debated for decades (reviewed in [1,2]). Here, we created 15 small (i.e. two mutational-step) fitness landscapes composed of three single amino acid substitutions at a gene involved in ABR and whole-gene knock-out mutations of five different genes. We screened these fitness landscapes at increasing gradients of temperature and an antibiotic. We found relatively little epistasis for four of the five different epistasis metrics. The benign environments exhibited the most epistasis, and adding antibiotic decreased epistasis (figure 3). ABR mutations, but not gene knock-outs, exhibited diminishing-returns epistasis in most environments (figure 4). These results suggest that, while epistatic interactions may confound predictions in the absence of an antibiotic, the effects of ABR mutations are predictable in the presence of an antibiotic.

### (a) Biological significance of our experiment

We combined ABR mutations with gene knock-out mutations because the presence or absence of accessory genes is the most common standing genetic variation in natural populations of *E. coli* [52]. To our knowledge, this is the first study to systematically investigate how G × G interactions between SNPs and gene deletions change across different environments. We selected the gene knock-outs especially to avoid direct, mechanistic interactions with *rpoB* or other genes involved in transcription, protein synthesis or DNA supercoiling. Therefore, our null expectation was that there would not be any G × G interactions and that fitness would be additive throughout. If there was specific epistasis as a result of direct, mechanistic interactions between the ABR mutations and gene knock-outs, we expected to observe masking epistasis where the effect of the complete gene deletion is dominant over all of the ABR mutations. We did observe masking epistasis, but in the opposite direction than expected under specific epistasis: the ABR mutations dominated over the knock-outs. This demonstrates that the effects of single amino acid substitutions at the *rpoB* locus are robust to non-core gene knock-out mutations. This result is an example of non-specific epistasis. Given its large population sizes and diverse pangenome composition, the core genes of *E. coli*, like *rpoB*, may have evolved to tolerate common polymorphisms like gene gain and loss events. We hypothesize that low pairwise epistasis between single amino acid substitutions in core genes and knock-out mutations of non-core genes may generally be expected for *E. coli* and other prokaryotes with large pangenomes [53].

We studied ABR mutations at the *rpoB* locus because this locus is clinically important and was shown to exhibit G × G interactions. Amino acid mutations at the *rpoB* locus (e.g. at site H526 included in our study) are the main, evolutionarily conserved mechanism of rifampicin resistance relevant for many bacterial pathogens [54,55]. This includes the clinically relevant *Mycobacterium tuberculosis*, against which rifampicin treatment remains an important first-line antibiotic [54]. Previous studies have found that the fitness of *rpoB* mutants depends on the presence of mutations in other mechanistically interacting proteins, like other subunits of the RNA polymerase complex [56] and other protein complexes involved in protein synthesis and DNA stability [31,57]. This type of ‘specific epistasis’ is easily explained by the close physical and functional proximity of these proteins to *rpoB*. ‘Non-specific epistasis’, however, has been observed between genes and proteins that do not interact directly [26]. *RpoB* is an excellent candidate to investigate non-specific epistasis as it was already shown to be highly pleiotropic: *rpoB* mutations impact the expression of hundreds of genes and many cell phenotypes [36,58–60]. The demonstrated functional effect of *rpoB* upon many other genes implies that mutations at other loci could impact the fitness of ABR *rpoB* mutations. Therefore, the quantification of G × G interactions at the *rpoB* locus is important to know how the danger of ABR depends on the genetic background.

We detected a signal of pairwise epistasis in only one of five gene knock-outs, Δ*waaP* (electronic supplementary material, figure S21). We had expected Δ*marR* to be the most likely knock-out mutation to exhibit pairwise epistasis, given its functional role in responding to chemical stressors and antibiotics [42,43,61] and its previously observed epistatic interactions with ABR mutations at genes involved in DNA stability [62]. Δ*marR* did not display significant pairwise epistasis in any environment; only Δ*waaP* did. The Δ*waaP* genotype on the susceptible *rpoB* wild-type background was more sensitive to low antibiotic concentration than the wild type and other knock-out single mutants, but this sensitivity was masked in combination with any of the three ABR mutations. Gene deletions of other lipopolysaccharide biosynthesis genes were shown to exhibit negative pairwise epistasis with ABR mutations [63]. Δ*waaP* could make cells more sensitive to rifampicin by increasing the permeability of the outer membrane [64,65]. Among the four functional genes studied, the *waaP* gene has the highest gene diversity (*π*_*waaP*_ = 0.034 versus *π*_*nuoC*_ = 0.015, *π*_*marR*_ = 0.014, *π*_*yidK*_ = 0.013) and is most frequently found knocked out (*waaP* is present in 78.5% of strains as compared to 99.5%, 99.3% and 90.8% for *nuoC, marR* and *yidK*, respectively) in natural populations, according to the panX database. Moreover, *waaP* impacts bacterial virulence and, so, is relevant in host–pathogen interactions [66]. *E. coli* strains from which *waaP* is absent may be more sensitive to low doses of rifampicin and other antibiotics than *waaP*-carrying genotypes. Our observed pattern of epistasis implies that this sensitivity is masked when the genotype carries a resistance mutation. Thus, the G × G interaction of *waaP* and *rpoB* erases selection against Δ*waaP* mutations that would occur at low doses of rifampicin.

### (b) Measurement challenges of gene-by-gene interactions

One methodological challenge for detecting G × G interactions in our study was that, in the presence of antibiotics, the fitness effects of the ABR mutants quickly overshadowed those of the knock-outs. In this case, methods that involve a relative comparison of direct fitness effects to epistatic effects are bound to infer less epistasis when there are stronger direct effects. The negative correlation of the roughness-to-slope ratio as a function of antibiotic concentration could be explained by this phenomenon: we observed mostly neutral fitness effects and moderate epistasis in the absence of the antibiotic but large beneficial fitness effects and no epistasis in the presence of the antibiotic. However, the negative correlation between antibiotic concentration and epistasis was also exhibited by other epistasis summary statistics that are independent of the size of direct fitness effects (i.e. gamma epistasis and the mean fraction of reciprocal sign epistasis, figure 3). Therefore, the observed decrease of epistasis with antibiotic concentration is likely a real biological phenomenon.

Our estimates of gamma epistasis for different gene classes suggest that the fitness landscape as a whole was less epistatic than when only ABR mutations or knock-out mutations were considered. This could reflect a biological pattern (as supported by the low pairwise epistasis results) where the epistatic interactions between amino acid mutations and gene knock-outs are smaller than within those mutational classes, perhaps due to evolved mutational robustness for gene knock-outs in *E. coli*. Nevertheless, the different trends observed between the gamma epistasis of the different mutational classes are possibly impacted by the differences in sample size. The gamma statistic relies on averaging over multiple mutational effects, which leads to a dilution of the epistatic signal for larger fitness landscapes. The total dataset has a larger sample size than subsets of the data, and the knock-out mutational class has a larger sample size than the ABR class. Future work should explore how the gamma epistasis measure can be used more appropriately for comparing epistasis between gene classes and differently sized fitness landscapes, for example, by randomly subsampling the data to an equal size for all categories.

### (c) Environmental interactions and their importance

It has long been established that there is a G × E interaction between rifampicin resistance mutations at the *rpoB* locus and temperature [67], among other environmental factors [18,68,69]. In a previous experiment, Rodríguez-Verdugo *et al.* [36] observed that *E. coli* adapted to high temperature evolved rifampicin resistance despite no rifampicin treatment. Therefore, we hypothesized that there would be an E × E interaction between high-temperature environments and rifampicin environments, which, if mediated by ABR mutations at the *rpoB* locus, could result in *G*_*rpoB*_ × *E*_AB_ × *E*_T_ interactions as well. Overall we observed no cost of rifampicin resistance in minimal media, which is consistent with Lin *et al.* [70] and could be attributed to ABR *rpoB* mutations mimicking the stringent response [56]. There was a modest positive effect of high temperatures on the competitive fitness of all genotypes and a weak E × E interaction between temperature and antibiotic. However, contrary to previous studies that also used batch cultures with glucose and minimal medium [7,36], the *rpoB* mutations S512F and I572S did not grow better and H526Y did not grow worse than the wild type at higher temperatures. (In fact, H526Y was observed to grow *better* than the wild type at 42°C.) The discrepancy for I572S could be attributed to G × G × E with the genetic background: Rodríguez-Verdugo *et al.* [36] found a strong effect of *E. coli* genetic background on the fitness at site I572. On the other hand, the discrepancies for S512F and H526Y could be that, unlike in Trindade *et al.* [7], our batch cultures were not acclimatized to the environment in which the competitions occurred. This underscores the sensitivity of organisms to their environments and the importance of complete reporting of experimental methods.

We used the overall environmental quality as a regressor to quantify the interaction between epistasis and environment, G × G × E. We found that the antibiotic concentration alone was a better regressor of epistasis than the environmental quality, implying that temperature did not have an effect on epistasis. We had expected that the overall environmental quality would be a better regressor because it is highly correlated with antibiotic concentration while also taking into account the effects of temperature. On the other hand, fitting of linear models with higher-order interactions suggested that temperature exhibited a stronger interaction with *G*_*rpoB*_ × *G*_KO_ than antibiotic concentration. Unfortunately, we have limited confidence in the linear regression results due to the low sample size and the high order of the investigated interactions. However, this apparent contradiction raises new questions about G × G × E interactions: for example, is it possible that one environmental variable affects direct G × E interactions, whereas the other acts on the G × G × E level? We are not aware of any previous work that has extracted such systematic patterns empirically, inferred their functional underpinnings, or developed models to account for such interaction. Certainly, the consequences of different interaction levels will be important to study in the future to predict potential evolutionary trajectories in varying environments.

### (d) Why is there so little epistasis in the studied antibiotic resistance fitness landscapes?

Overall, we found little G × G and G × G × E interaction in our data. This result is in stark contrast to various recent experimental studies, which have presented strong evidence that both fitness effects of single mutations and their epistatic fitness interactions may vary greatly between environments ([9,10,19], reviewed in [2]). One reason for this discrepancy may be the choice of mutations for the fitness landscapes. Previous studies focused on SNPs between or within genes that had been found in an adaptive walk [4,5,11], indirectly inferred strong G × G(×E) by observing that adaptations were unique to the genetic background [21,30,71], or measured epistasis as the different effect of ABR mutations across vastly different genetic backgrounds [72,73]. We here quantified epistatic interactions between ABR SNPs and (non-ABR) gene knock-out mutations that occur in natural populations of *E. coli*. The studied combinations of mutations thus had no immediate relationship except the *rpoB* mutations’ previously characterized G × E interaction for fitness under higher temperature or antibiotic. However, multiple studies in systems or molecular biology have shown that epistasis is common (reviewed in [26]). In particular, when two or more beneficial mutations are combined, negative epistasis in the shape of diminishing returns (i.e. where the fitness effect of a mutation is less beneficial when it occurs on a fitter background) has been observed ubiquitously [28,29,63,74], including for *rpoB* mutations conferring ABR to rifampicin [75]. In our study, we observed diminishing-returns epistasis only for the *rpoB* ABR mutations but no diminishing-returns epistasis for the knock-out mutations. Moreover, we did not observe any qualitative environmental trend in the strength of the diminishing-returns epistasis for the ABR mutations. However, the mutations shifted from neutral to beneficial as a function of antibiotic. Although there have been studies that derived null expectations of epistasis between random mutations of the same type [76], between mutations characterized by their fitness effect [77], or between SNPs in a pair of genes in a metabolic pathway [78], we are not aware of any general models or empirical studies that have proposed or quantified a null distribution of epistatic effects between SNPs and different structural mutation types (except for the assumption of no epistasis). Therefore, it is not clear whether the low G × G and G × G × E interactions observed in our data are to be expected.

Despite a lack of general epistatic null models, theoretical and empirical works have proposed a few hypotheses on how much epistasis to expect between mutations that confer ABR. For example, Mira *et al.* [34] found ubiquitous sign epistasis, in all 30 environments assayed, for a fitness landscape with all possible combinations of four ABR mutations. Knopp & Andersson [31] found that epistasis was rare between combinations of ABR mutations. Our finding that ∼20% of randomly selected combinations of mutations exhibit pairwise epistasis is in good quantitative agreement with the results of Knopp & Andersson [31]. A critical difference between our work and most previous work on ABR epistasis is that the knock-out mutations we investigated neither interacted directly with *rpoB* (as required for specific epistasis) nor, except Δ*waaP*, displayed any significant effects on fitness (electronic supplementary material, figure S28). Epistasis expressed by seemingly neutral mutations is termed ‘cryptic epistasis’, and has been previously observed between mutations that were fixed during an adaptive walk [3,5]. We have uncovered new instances of cryptic epistasis that depend on the environment. Using a theoretical model, Engelstädter [79] related the expected epistasis to the extent of G × E interactions of the involved mutations: they proposed that when there is no cost to the ABR mutations, there should be no epistasis between them. Moreover, a study by Das *et al.* [13], considering only ABR mutations with a cost of resistance predicted using mathematical models and empirically, observed the strongest epistasis at intermediate antibiotic concentrations. Our experiment screened a range of intermediate antibiotic concentrations, with 4 μg ml^−1^ below and 10 μg ml^−1^ near the minimum inhibitory concentration (MIC) of the wild-type *rpoB* (electronic supplementary material, figure S14), and ABR mutations without a cost of resistance (although a cost had been expected according to previous studies, see above) in combination with non-ABR mutations. Our finding of very little epistasis in the presence of antibiotic is thus more consistent with the prediction of Engelstädter [79].

### (e) Do fitness landscapes become smoother as the concentration of an environmental stressor increases?

How generalizable are our findings that fitness landscapes become smoother in more stressful environments? To discuss this question, we compare our findings with those of Gorter *et al.* [8], who measured fitness under different cadmium concentrations for all combinations of knock-out mutations that had evolved in response to those toxic environments. Contrary to our results, their co-selected mutations conferring resistance to increasing cadmium exhibited increasingly strong selective effects and positive pairwise epistatic effects as the heavy-metal concentration increased [8]. Interestingly, both our results and those of Gorter *et al.* [8] contradict theoretical predictions. According to Fisher’s geometric model [80], the average pairwise epistasis should be unchanged with environmental stress for random mutations, like the combinations of mutations used in our study, whereas it should decrease for co-selected mutations, like those studied in Gorter *et al.* [8]. Also, our results and those of Gorter *et al.* [8] differ from the predicted and empirical results of Das *et al.* [13] that epistasis should be strongest at an intermediate concentration of an antibiotic stressor. However, the model of Das *et al.* [13] was specific to increasing antibiotic (and not cadmium) stress and assumed a cost of resistance which was not observed in our study or in Gorter *et al.* [8]. These conflicting results and predictions call for the study of additional empirical fitness landscapes under increasing concentrations of an environmental stressor, and new theoretical fitness landscape models that incorporate mechanistic details of biological phenomena.

Our main conclusion is that epistasis decreases with increasing antibiotic concentration. Only in the absence of the antibiotic, we observed several instances of reciprocal sign epistasis for all three ABR mutations and at all temperature environments (figure 3*b*; electronic supplementary material, table S7). The presence of reciprocal sign epistasis implies that the fitness landscape has multiple peaks and that evolution may be less predictable in the absence of antibiotic. Overall, our results are consistent with the conclusion that the underlying fitness landscapes of ABR mutations and gene knock-outs are more rugged in the absence of antibiotic than in the presence of higher concentrations of antibiotic. Extrapolated to the larger sequence space, our results would imply that evolution is more predictable in the presence than in the absence of antibiotic, because ABR mutations have such strong beneficial effects in the presence of high antibiotic concentrations. Here, the strong effect of the ABR mutations potentially overrides any effects of the genetic background.

## Data Availability

All flow cytometry data, competitive fitness estimates and the annotated code used to generate the analyses are publicly available at the following Git repository: https://gitlab.com/evoldynamics/epistasis-decreases-with-increasing-antibiotic-pressure and is archived on Zenodo with the following doi:10.5281/zenodo.7661199. The whole-genome sequencing is archived on NCBI with the BioProject accession: PRJNA910115. The data are provided in electronic supplementary material [81].
